# Immunoreactivities Against Different Tyrosine-Phosphatase 2 (IA-2)(256-760) Protein Domains Characterize Distinct Phenotypes in Subjects With LADA

**DOI:** 10.3389/fendo.2022.921886

**Published:** 2022-06-24

**Authors:** Claudio Tiberti, Luca D’Onofrio, Francesca Panimolle, Simona Zampetti, Ernesto Maddaloni, Raffaella Buzzetti

**Affiliations:** Experimental Medicine Department, Sapienza University of Rome, Rome, Italy

**Keywords:** diabetes, autoantibodies, IA-2 antibodies, tyrosine-phosphatase 2, obesity, LADA

## Abstract

Antibodies (Abs) against intracellular epitopes of the tyrosine-phosphatase 2 (IA-2) are detected in type 1 diabetes. Abs directed against the IA-2(256-760) portion, with both intra- and extracellular epitopes, are present in people with latent autoimmune diabetes in adults (LADA) and in obese subjects with normal glucose tolerance (NGT). We aim to characterize distribution and clinical features of intra- and extra-cellular IA-2(256-760) immunoreactivities in people with LADA compared to obese people with NGT. The intracellular immunoreactivity represented by immune response against two intracellular IA-2 constructs (IA-2JM(601-630) and IA-2IC(605-979)) was analyzed and related to clinical and biochemical features in 101 people with LADA and in 20 NGT obese subjects, all testing positive for IA-2(256-760) Abs. IA-2 intracellular immunoreactivity showed a frequency of 40.6% in LADA while it was not detected among NGT obese (p<0.001). Amongst LADA, the presence of immunoreactivity against the IA-2 intracellular domains was associated with lower BMI, waist circumference, higher HDL cholesterol and lower triglycerides, lower prevalence of hypertension and higher prevalence of other autoimmune disorders. Immunoreactivity against IA-2 does not involve intracellular domains in the majority of LADA and in obese people with NGT. This study shows that there is heterogeneity in the IA-2 epitopes, associated with different clinical features.

## Introduction

Tyrosine-phosphatase 2 protein (IA-2) is one of the major autoantigens in autoimmune diabetes, target of both humoral and T-cell reactivity and thus representing a molecule of potential pathogenic significance (1, 2). The spreading of autoimmune response within and between islet pancreatic autoantigens is crucial for disease progression (3). In particular, the presence of immunoreactivity against multiple IA-2 epitopes in subjects with prediabetes increases the risk of the disease (4). Indeed, several regions of IA-2 may be target of immunoreactivity (5–9). In classical type 1 diabetes (T1D), IA-2 autoantibodies (Abs) are frequently directed against multiple epitopes of the intracellular cytoplasmic domain of the protein (a.a.601-979), including the juxtamembrane region (JM) and the PTP-like C-terminal domains (3, 10–12). Conversely, the immunoreactivity in people with adult-onset autoimmune diabetes not requiring insulin at diabetes onset was mainly directed against the IA-2_(256-760)_ portion of the protein, which contains both intra- and extracellular domains (5). We have previously demonstrated that latent autoimmune diabetes in adults (LADA) subjects positive for IA-2_(256–760)_ but negative for GAD Abs showed a phenotype resembling classical type 2 diabetes (T2D), with higher BMI, waist circumference, and lower progression to insulin requirement than those positive for both IA-2_(256–760)_ and GAD Abs (13). This finding underlined the great heterogeneity among LADA subjects positive for IA-2_(256-760)_ Abs. In addition, IA-2_(256-760)_ Abs were the most prevalent form of immunoreactivity detected in LADA subjects with a BMI >30 kg/m^2^ (13, 14). Notably, IA-2_(256-760)_ Abs were also detected in 3.2% of people with obesity but without diabetes (15). As a result, the evidence published so far indicates that IA-2_(256-760)_ protein intercept immunoreactivities in different metabolic conditions (classical T1D, LADA and even obesity without diabetes). We hypothesized that this might be due to the presence in its domain of both intra- and extracellular aminoacidic sequences, which might be targeted differently in people with different clinical and autoimmune features.

**Figure 1:**
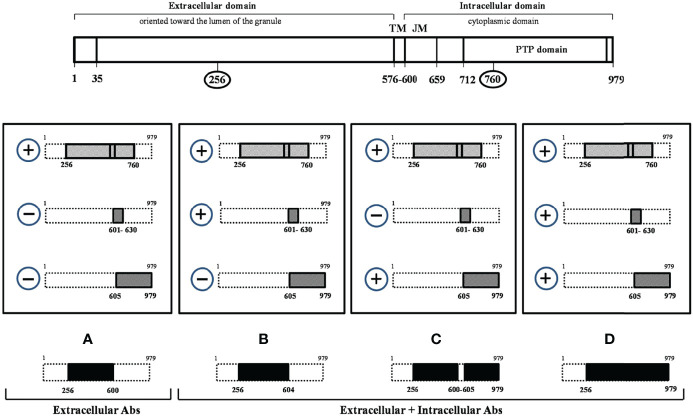
Tyrosine phosphatase 2 (256-760) immunoreactivity dissection study. At the top of the figure the tyrosine phosphatase 2 (IA-2) full-length protein (a.a.1-979) is schematically represented. The four rectangles in the central part of the figure indicate all possible combinations resulting from the analysis of IA-2(256-760) Ab positive sera with IA-2JM(601-630) and IA-2IC(605-979) constructs. In the lower part of the figure the IA-2 domains **(A–D)** resulting by the various patterns of immunoreactivity and that distinguish the extracellular alone from more complex forms of immunoreactivity against IA-2 protein are shown in black.

Therefore, in this study we aimed at evaluating whether the aminoacidic sequence recognized by IA-2_(256-760)_ Abs differ between IA-2_(256-760)_ Abs positive LADA and obese people with normal glucose tolerance (NGT), and to relate different immunoreactivity patterns to metabolic features. In particular, we tested the immunoreactivity against two IA-2 constructs, IA-2_JM(601-630)_ and IA-2_IC (605-979)_, which account for the whole IA-2 intracellular domain immunoreactivity. This evaluation could allow us to identify whether the positivity for IA-2_(256-760)_ was due to the immunoreactivity towards the extra or intracellular portion of IA-2 protein in the two groups of subjects (**Figure 1**).

## Research Design and Methods

### Study Design and Population

In this cross-sectional study conducted at the Department of Experimental Medicine, “Sapienza” University of Rome, we analyzed in LADA (n=101) and obese people with NGT (n=20) positive for IA-2_(256-760)_ Abs, the immunoreactivity against two IA-2 constructs, namely IA-2_JM(601-630)_ and IA-2_IC (605-979)_, evaluating the relative clinical features. For the aims of the study, we had not a disposal a single construct including only the intracellular domain of IA-2 protein (a.a. 601-979), thus we utilized IA-2_JM(601-630)_ and IA-2_IC (605-979)_ that overlap to a small extent, but altogether account for the whole intracellular domain of IA2-protein. More specifically people with LADA and NGT obese subjects were enrolled among patients attending the Diabetes Unit of Umberto I General Hospital. In order to reduce any possible selection bias, consecutively subjects with LADA or obese people with NGT attending the outpatients clinics were screened for IA-2(256-760) Abs. In particular, considering that the prevalence of IA-2(256-760) Abs is around 3.5% in people with T2D (13) and 3.00% in obese population without diabetes (15), we have screened approximately 3000 subjects with T2D to found subjects with LADA positive for IA-2(256-760) Abs and approximately 600 obese subjects without diabetes.

For this study LADA was defined in the presence of the following criteria 1) a diagnosis of diabetes mellitus according to the criteria proposed by the American Diabetes Association (16); 2) age at diabetes onset >30 years; 3) insulin independence for at least 6 months after diabetes diagnosis; 4) evidence of pancreatic autoimmunity as testified by the presence of at least one autoantibody among GAD Abs and IA-2 Abs. Obese people with NGT were defined by the presence of a BMI ≥30 Kg/m^2^, fasting blood glucose values <110mg/dL, HbA1c <6.0% and normal blood glucose values after an oral glucose tolerance test (<140mg/dL after 2 hours).

### Data Collection

The following clinical and biochemical data were collected from people with LADA and from NGT obese subjects: age, age at diagnosis of diabetes for LADA, waist circumference, fasting blood glucose, HbA1c, total cholesterol, HDL cholesterol and triglycerides. LDL cholesterol concentrations were estimated with the Friedewald formula: total cholesterol – [HDL + (Triglycerides/5)], history of hypertension and presence of other autoimmune diseases.

### Evaluation of Immunoreactivity

Abs against IA-2_(256-760)_ (14), IA-2_JM(601-630)_ (5), IA-2_IC(605-979)_ (8) and GAD (5) were measured by previously described radioimmunoprecipitation assays, using the corresponding *in vitro* translated [^35^S]-methionine labelled proteins. Human recombinant IA-2(256-760) cDNA was provided by Dr. Liping Yu (University of Colorado, Aurora, Denver, USA). Human recombinant IA-2_IC(605-979)_ and IA-2_JM(601-630)_ cDNAs were provided by Dr Ezio Bonifacio (University of Dresden, Dresden, Germany). The positive autoantibody indexes for IA-2_JM(601-630)_ and IA-2_IC(605-979)_ constructs were defined as values >99th percentile of 211 healthy control sera (5). The specificity and sensitivity of IA-2_JM(601-630)_ were 99% in 100 healthy controls and 9.6% in GAD-autoantibody positive LADA patients, respectively. The specificity and sensitivity of IA-2_IC(605-979)_ were 99% in 100 healthy controls and 19.8% in GAD-autoantibody positive LADA patients, respectively. This last construct is the one that we currently use in our previous study on autoimmune diabetes and whose performance has been evaluated along the time in several Diabetes Autoantibody Workshops (i.e. 99% specificity and 72% sensitivity at the Fourth DASP). The evaluation of immunoreactivity for three different IA-2 epitopes (IA-2_(256-760)_, IA-2_JM(601-630)_ and IA-2_IC(605-979)_ fragments) allowed to identify whether the immunoreactivity against the IA-2_(256-760)_ was limited to the extracellular domain of the protein or involved also its intracellular domain. **Figure 1** shows a schematic representation of the full-length IA-2 protein (a.a.1-979) and of the three IA-2 clones utilized in the study (IA-2_(256-760)_, IA-2_JM(601-630)_, IA-2_IC(605-979)_).

In people with confirmed immunoreactivity against IA-2_(256-760)_ Abs, as those enrolled in this study, four mutually exclusive patterns of immunoreactivity can be obtained by measuring Abs against IA-2_JM(601-630)_ and IA-2_IC(605-979)_ constructs. Among these four patterns only pattern A (IA-2_(256-760)_ Ab positive, but IA-2_JM(601-630)_ and IA-2_IC(605-979)_ negative patients) identifies an immunoreactivity limited to the extracellular IA-2 domain only.

Human recombinant full-length GAD-65 cDNA was provided by Dr Åke Lernmark (Lund University, Malmoe, Sweden).

### Statistical Analysis & Ethics

Descriptive statistics are presented for categorical variables as numbers with proportions, and for continuous variables as appropriate measures of central tendency and dispersion. The Shapiro-Wilk test was used to test the normality distributions of variables examined. T-test or ANOVA were used to test differences of continuous variables with parametric distribution between two or more groups, respectively, while Mann-Whitney and Kruskall-Wallis tests were used for non-parametric variables. Categorical variables were compared with a χ² or Fisher’s exact test as appropriate. Two-sided tests at the 0.05 level of significance were used for all statistical comparisons. IBM SPSS Statistics 21 software was used for data analysis and Prism 9 software was used for graphical representations.

The study was performed in accordance with the Declaration of Helsinki, and the study procedures were approved by the institutions’ ethics committees (Prot. 287/2020, ref 5984).

## Results

### Population Features

Clinical feature of people with LADA and obese subjects with NGT are summarized and compared in **Table 1**. Briefly, people with LADA were more frequently males (55.4% vs 20.0%, p=0.003), older (55.2 [48.5-61.2]years vs 43.5 [35.3-54.3]years, p=0.003) and leaner (BMI: 27.9[24.4-32.1]Kg/m^2^ vs 39.5[36.7-44.1]Kg/m^2^, p<0.001; waist circumference: 95[87-106]cm vs 123[115-129]cm, p<0.001) compared to NGT obese subjects. No significant differences were found in the lipid profile, and in the prevalence of hypertension and of other autoimmune diseases. As expected, fasting blood glucose and HbA1c values were higher among people with LADA.

**Table 1:** Characteristics of LADA and NGT obese subjects enrolled in the study. All subjects reported in the table were positive for IA-2_(256-760)_ Abs.

	Obese, n=20	LADA, n=101	p
Age, years	43.5 [35.3-54.3]	55.2 [48.5-61.2]	**0.003**
Disease duration, years	NA	2.0 [0.4-3.9]	
BMI, kg/m2	39.5 [36.7-44.1]	27.9 [24.4-32.1]	**<0.001**
Waist circumference, cm	123 [115-129]	95 [87-106]	**<0.001**
HbA1c, %	5.5 [5.3-5.7]	7.2 [6.2-8.3]	**<0.001**
HbA1c, mmol/mol	37 [34-39]	55 [44-67]	**<0.001**
Fasting blood glucose, mg/dL	86 [83-106]	136 [116-169]	**<0.001**
Total cholesterol, mg/dL	197 [161-254]	184 [150-226]	0.286
LDL, mg/dL	121 [88-161]	106 [87-141]	0.247
HDL, mg/dL	49 [39-56]	45 [38-56]	0.893
Triglycerides, mg/dL	132 [90-168]	145 [91-196]	0.578
GAD Abs + (n), %	0	(46) 46.0%	**<0.001**
Gender, male (n), %	(4) 20%	(56) 55.4%	**0.003**
Hypertension prevalence (n), %	(10) 50.0%	(45) 44.6%	0.064
Other autoimmune diseases (n), %	(2) 10.0%	(22) 21.8%	0.176

Significant p-values are provided in bold.

### Immunoreactivities Against Intra- and Extra-Cellular IA-2 Domains

**Figure 2:**
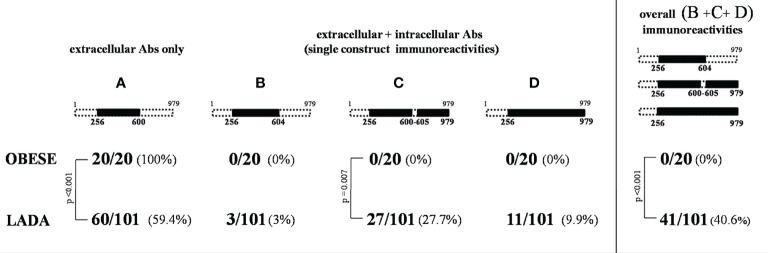
IA-2 patterns of **(A–D)** immunoreactivity in subjects with LADA and NGT obese subjects.

A significantly different distribution of the four IA-2 immunoreactivity patterns was found between people with LADA and obese subjects with NGT (p=0.006) (**Figure 2**). In particular, pattern A (immunoreactivity limited to the extracellular IA-2 domain) was found in 59.4% of people with LADA, while it was the only pattern found in obese subjects with NGT, who did not show any marker of immunoreactivity against the intracellular domains of IA-2 (p<0.001 for the difference in the prevalence of pattern A between LADA and NGT obese subjects).

### Population Features According to Patterns of IA-2 Immunoreactivity

In **Table 2** the clinical features of obese subjects with NGT compared to people with LADA showing pattern B+C+D or absence of immunoreactivity against the intracellular IA-2 domains (pattern A) have been reported.

**Table 2:** Clinical characteristics of NGT obese subjects and of people with LADA according to different patterns of Immunoreactivity.

	NGT obese: IA-2 extracellular immunoreactivity only, n=20	LADA: with IA-2 extracellular immunoreactivity only (A), n=60	LADA: showing intracellular immunoreactivities (B+C+D), n=41	p *
Age, years	43.5 [35.3-54.3]	56.5 [51.1-65.5]	53.5 [40.0-57.5]^‡^	**<0.001**
Age at onset, years	n.a.	54.2 [49.9-60.7]	50.7 [38.4-54.3]^‡^	
Disease duration, years	n.a.	2.5 [0.2-4.0]	1.5 [0.8-3.0]	
BMI, Kg/M^2^	39.5 [36.7-44.1]	29.4 [26.4-32.7]^‡^	26.0 [22.7-30.0]^§‡^	**<0.001**
Waist circumference, cm	123 [115-129]	101 [91-111]^‡^	92 [84-100]^§‡^	**<0.001**
HbA1c, %	5.5 [5.3-5.7]	6.8 [6.3-8.1]^‡^	7.5 [6.2-9.5]^§^	**<0.001**
HbA1c, mmol/mol	37 [34-39]	51 [45-65]^‡^	58 [44-8]^§^	**<0.001**
Fasting blood glucose, mg/dL	86 [83-106]	130 [115-160]^‡^	150 [118-194]^§||^	**<0.001**
Total cholesterol, mg/dL	197 [161-254]	191 [151-233]	183 [149-217]	0.413
LDL, mg/dL	121 [88-161]	109 [87-143]	104 [88-140]	0.460
HDL, mg/dL	49 [39-56]	42 [35-52]	48 [43-62]^‡^	**0.02**
Triglycerides, mg/dL	132 [90-168]	175 [106-218]	101 [73-181]^‡^	**0.015**
GAD Abs + (n), %	0	(8) 13.6%	(38) 92.7%^¶^	**<0.001**
Gender, male (n), %	(4) 20%	(25) 42.4%	(19) 46.3%	**0.012**
Hypertension (n), %	(10) 50.0%	(34) 57.6%	(11) 26.8%^‡^	**0.002**
Other autoimmune diseases (n), %	(2) 10.0%	(7) 14.6%	(15) 57.7%^¶^	**<0.001**

*P-value for the difference among the three groups.

^‡^ only LADA with only IA-2 extracellular immunoreactivity (A) Vs NGT obese p<0.001;

**^§^** only LADA showing intracellular immunoreactivities (B+C+D) Vs NGT obese p<0.001;

**^||^** only LADA with only IA-2 extracellular immunoreactivity (A) Vs LADA showing intracellular immunoreactivities (B+C+D) p=0.05;

**^‡^** only LADA with only IA-2 extracellular immunoreactivity (A) Vs LADA showing intracellular immunoreactivities (B+C+D) p<0.01;

**^¶^** only LADA with only IA-2 extracellular immunoreactivity (A) Vs LADA showing intracellular immunoreactivities (B+C+D) p<0.001. Significant p-values are provided in bold.

People with LADA and IA-2 immunoreactivity limited to the extracellular domain of the protein (pattern A) were older (p<0.01), with higher age at the time of diagnosis (p<0.01), higher BMI (p<0.001), higher waist circumference (p<0.01), lower HDL cholesterol levels (p<0.01), higher triglycerides levels (p<0.01), higher prevalence of hypertension (p<0.01) and lower prevalence of other autoimmune disorders (p<0.001) compared to people with LADA showing also intracellular immunoreactivity (pattern B+C+D).

Nonetheless, obese people with NGT still showed the highest BMI and waist circumference values compared to both LADA with and without intracellular immunoreactivity (p<0.001 for all trends and pairwise comparisons).

Finally, analyzing only subjects with LADA, a significant difference in BMI was found stratifying subjects according to the four IA-2 immunoreactivity patterns (group A 29.4[26.4-32.7]kg/m2 Vs group B 28.1[23.1-]kg/m2 Vs group C 26.0[22.3-30.8]kg/m2 and group D 25.0[23.2-28.3]kg/m2, p=0.038) as shown in **Supplementary Figure 1**.

Further, evaluating treatment for diabetes, no differences in oral hypoglycemic treatment (respectively 91% Vs 89%, p=0,831) or insulin treatment (respectively 18% Vs 6%, p=0.1) was found between LADA with and without intracellular immunoreactivity.

### Frequencies of GADA According to Different Patterns of Immunoreactivity in LADA

GAD Abs were not found in the obese subjects with NGT, while GAD Abs positivity was detected in 46% of people with LADA (p<0.001). Among people with LADA, GAD Abs were less frequently found in subjects showing IA-2 immunoreactivity limited to the extracellular portion of the protein (pattern A) compared to those testing positive for Abs also against the intracellular aminoacidic sequences (13.6% vs 92.7%, p<0.001) (**Table 2**).

## Discussion

This study shows that there is a heterogeneity in the epitope domains recognized by IA-2_(256-760)_ Abs and that distinct domains are related to different clinical and metabolic features of LADA and obese patients. Specifically, our study suggests that only the extracellular portion of the IA-2 protein works as an immune-domain in obese people with NGT as well as in the large subgroup of people with LADA characterized by clinical features closer to T2D than to classical T1D. Conversely, immunoreactivity against the IA-2 intracellular aminoacidic sequences appears more frequently in people with a clearer autoimmune clinical profile characterized by low BMI, presence of GADA, multiple autoimmune disorders.

IA-2 is a large transmembrane glycoprotein involved in the biogenesis and turnover of insulin secretory granules in pancreatic β-cells (17, 18) and known to be a major autoantigen in autoimmune diabetes. It could be speculated that extracellular portion of IA-2 could be accounted for an early target of autoimmune process towards pancreatic islet inflammation due to immunoreactivity process or low grade inflammation. To our knowledge there is no direct evidence of this phenomenon, but several data suggest a role for IA-2 protein. Previous studies showed that epitopes included in the 256-760 domain intercept immunoreactivity in different metabolic conditions. IA-2_(256-760)_ Abs were indeed detected in about one-third of subjects with pre-T1D who will progress towards the disease, in almost half people with T1D at the time of diagnosis (5, 8), in part of people with LADA (13, 19) and even in a small percentage of obese subjects with NGT (15). Nonetheless, the presence of IA-2_(256-760)_ Abs in people with diabetes without other pancreatic Abs was shown to identify patients with clinical phenotypes that resemble classic T2D and with a slower progression towards insulin therapy than patients who were GAD Abs positive (13). This suggests that the large 256-760 domain contains several epitopes associated with different autoimmune clinical conditions. Specifically, the 256-760 domain includes both intracellular and extracellular aminoacidic sequences, and in this study, by testing IA-2_(256-760)_ Abs positive sera with IA-2_JM(601-630)_ and IA-2_IC(605-979)_ constructs, we were able to identify and characterize people with immunoreactivity limited only to the extracellular portion of the IA-2 protein. We found that these subjects were obese people with NGT or LADA subjects more likely affected by central adiposity and hypertension, less likely affected by other autoimmune disorders and with a worse lipid profile than people with LADA showing also intracellular immunoreactivity. This evidence confirms the hypothesis that different metabolic phenotypes may be associated with the development of autoimmunity against different epitope domains and that the heterogeneity of autoimmune diabetes may rely on different types of immune dysregulation (20, 21). In particular, the strict association between the immunoreactivity limited to the extracellular portion of the IA-2 protein and an obese phenotype might support a role for low-grade inflammation in developing an autoimmune process directed against the β-cell, which is, however, different from what is observed in lean people (22). It is worth noting that the differences in clinical features of people target only of extracellular immunoreactivity and people with both extra- and intracellular immunoreactivity, resemble differences observed in subjects with LADA with high and low GAD Abs levels (23).

Longitudinal studies should be conducted to evaluate whether the extracellular IA-2 immunoreactivity associated to low-grade inflammation is then able to trigger a “spreading” of autoimmunity to intracellular epitopes, causing a more severe autoimmune disease in predisposed subjects or in the presence of precipitating factors.

A limitation of this study is that no data on HLA class II or other genetic features were available. Unfortunately, we do not have available information about islet cell function, such as C-peptide level. Nevertheless, this study has also several strengths, such as a well-characterized population of obese people with NGT and with LADA, both positive for IA-2_(256–760)_ Abs, and a detailed immunoreactivity panel towards different epitopes within the IA-2 protein.

In conclusion, this study shows that immunoreactivity against IA-2 is limited to the extracellular portion of the protein in obese people with NGT and in the majority of people with LADA. Heterogeneity in the IA-2 epitopes exists and it is associated with different clinical and metabolic features. Prospective studies are needed to confirm these findings especially evaluating the clinical course in subjects positive only for IA-2 extracellular immunoreactivity.

## Data Availability Statement

The raw data supporting the conclusions of this article will be made available by the authors, without undue reservation.

## Ethics Statement

The studies involving human participants were reviewed and approved by Policlinico Umberto I ethics committees. The patients/participants provided their written informed consent to participate in this study.

## Author Contributions

CT designed the study, conducted laboratory analysis and write the paper, LD’O designed the study, collected data and write the manuscript, FP and SZ conducted laboratory analysis, EM reviewed/edited the manuscript, RB contributed to discussion and reviewed manuscript. RB is the guarantor of this work and, as such, had full access of the data of the study and takes the responsibility of the integrity of the data and the accuracy of the data analysis. All authors contributed to the article and approved the submitted version.

## Funding

This study was funded by Italian Ministry of University and Research (PRIN 2017: 20175L9H7H)

## Conflict of Interest

The authors declare that the research was conducted in the absence of any commercial or financial relationships that could be construed as a potential conflict of interest.

## Publisher’s Note

All claims expressed in this article are solely those of the authors and do not necessarily represent those of their affiliated organizations, or those of the publisher, the editors and the reviewers. Any product that may be evaluated in this article, or claim that may be made by its manufacturer, is not guaranteed or endorsed by the publisher.
