# Supplementary material for: Immunoreactivities Against Different Tyrosine-Phosphatase 2 (IA-2)(256-760) Protein Domains Characterize Distinct Phenotypes in Subjects With LADA
Source: Front Endocrinol (Lausanne). 2022 Jun 24;13:921886. doi: 10.3389/fendo.2022.921886 (PMC9263087; doi:10.3389/fendo.2022.921886)

Supplementary figure 1. BMI levels in subjects with LADA patients subdivided by IA-2 A, B, C and D immunoreactivity patterns.

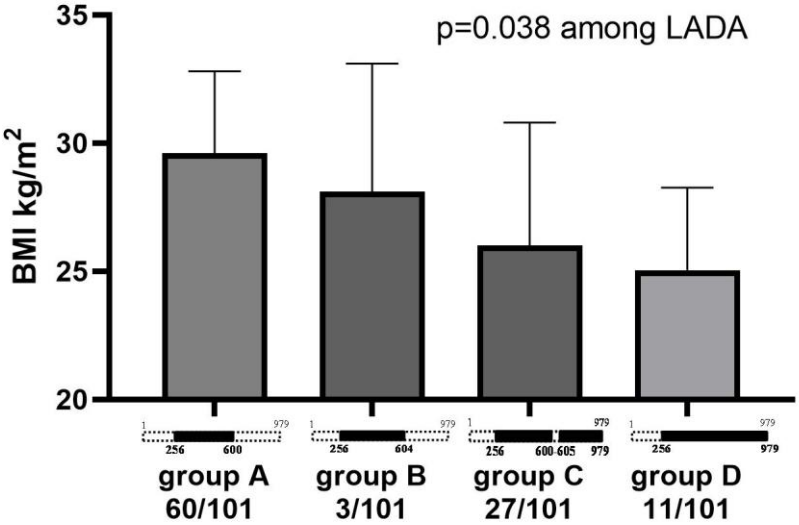

Supplement: Supplementary file 1 [file DataSheet_1.pdf]
